# Ultrasonography for Surgical Planning and Follow-Up in Neurofibromatosis Type 1

**DOI:** 10.3390/diagnostics16101556

**Published:** 2026-05-20

**Authors:** Po-Yin Shen, Cheng-Jung Ho, Wei-Ting Wu, Ke-Vin Chang, Levent Özçakar

**Affiliations:** 1 Department of Orthopaedics, Kaohsiung Municipal Siaogang Hospital, Kaohsiung 812, Taiwan; vanness068@gmail.com; 2 Department of Orthopaedics, Kaohsiung Medical University Hospital, Kaohsiung 807, Taiwan; rick_free@mail2000.com.tw; 3 School of Medicine, College of Medicine, Kaohsiung Medical University, Kaohsiung 807, Taiwan; 4 Department of Physical Medicine and Rehabilitation, Community and Geriatric Research Center, National Taiwan University Hospital, Bei-Hu Branch, Taipei 108206, Taiwan; wwtaustin@yahoo.com.tw; 5 Department of Physical Medicine and Rehabilitation, College of Medicine, National Taiwan University, Taipei 100233, Taiwan; 6 Center for Regional Anesthesia and Pain Medicine, Wang-Fang Hospital, Taipei Medical University, Taipei 110301, Taiwan; 7 Department of Physical and Rehabilitation Medicine, Hacettepe University Medical School, Ankara 06100, Turkey; lozcakar@yahoo.com

**Keywords:** neurofibroma, ultrasound, peripheral nerve sheath tumor

## Abstract

Ultrasonography can assist in the preoperative evaluation and postoperative surveillance of superficial soft tissue tumors of the hand. We present an ultrasound-based identification of a neurofibroma in a patient with neurofibromatosis type 1 (NF1). A 45-year-old male presented with a slowly enlarging subcutaneous mass over the dorsal aspect of the hand associated with localized paresthesia. Physical examination revealed characteristic NF1 stigmata, including café-au-lait macules, axillary freckling, and craniofacial asymmetry suggestive of sphenoid wing dysplasia. High-resolution ultrasonography demonstrated a well-defined hypoechoic fusiform lesion along the course of a digital nerve, suggestive of a peripheral nerve sheath tumor. Magnetic resonance imaging showed a T2-hyperintense lesion compatible with a nerve sheath tumor. Surgical excision was subsequently performed, and histopathological examination confirmed a localized neurofibroma with incorporation of native nerve fascicles within a myxoid spindle cell matrix. Serial postoperative ultrasonography at 3 and 12 months demonstrated no evidence of local recurrence. This case highlights ultrasonography as a practical, radiation-free, and cost-effective modality for both preoperative assessment and longitudinal follow-up of superficial NF1-associated neurofibromas.

**Figure 1:**
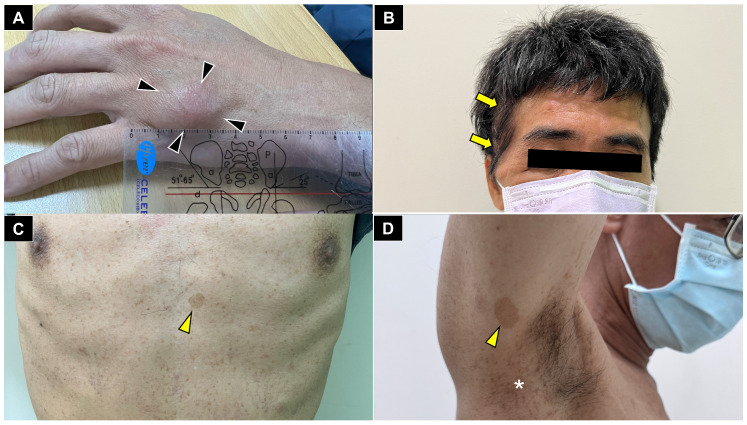
A 45-year-old male presenting with concurrent cutaneous and skeletal manifestations of neurofibromatosis type 1 (NF1). (**A**) Clinical photograph demonstrating a firm, ovoid subcutaneous mass (black arrowheads) located between the fourth and fifth metacarpals, approximately 3.5 cm in size. The patient reported a three-year history of gradual enlargement with recent onset of perilesional paresthesia. (**B**) Oblique craniofacial view demonstrating a visible bony contour depression over the right temporal region (yellow arrows). In the context of a childhood history of ophthalmologic surgery, this asymmetry raises suspicion for NF1-associated sphenoid wing dysplasia, a recognized osseous criterion under the revised diagnostic framework [1,2]. (**C**) Anterior trunk photograph demonstrating multiple hyperpigmented macules, including a representative café-au-lait macule exceeding 15 mm in greatest diameter (yellow arrowhead). (**D**) Right axillary region demonstrating a café-au-lait macule (yellow arrowhead) and intertriginous freckling consistent with the Crowe sign (white asterisk) [1,2]. Collectively, this patient fulfilled the 2021 revised international consensus criteria for NF1 [1] on the basis of multiple independent features: (1) six or more café-au-lait macules, (2) intertriginous freckling, (3) characteristic osseous involvement consistent with sphenoid wing dysplasia, and (4) a first-degree relative with confirmed diagnosis of NF1. Although molecular genetic confirmation was unavailable due to institutional resource limitations, the constellation of clinical findings was sufficient to establish a definitive clinical diagnosis of NF1 [1,2]. Preoperative brain MRI further excluded intracranial neurofibromas and optic pathway gliomas, addressing the right temporal depression and ophthalmic history. This case underscores the importance of systematic whole-body inspection when evaluating an apparently isolated soft tissue mass of the hand.

**Figure 2:**
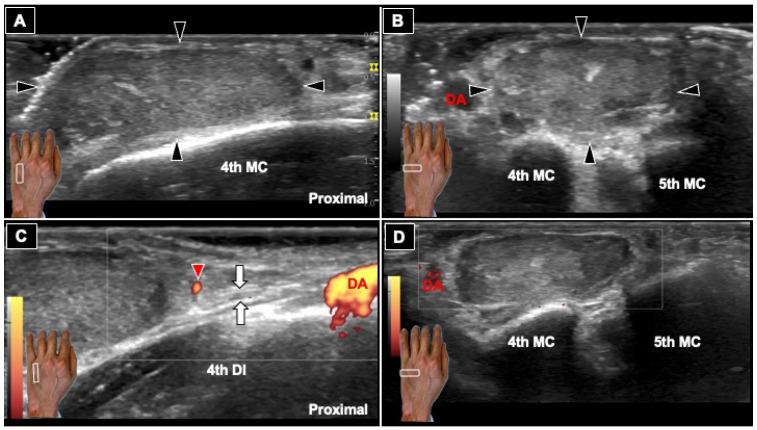
Ultrasonographic Evaluation of a Dorsal Hand Mass. (**A**) Long-axis B-mode ultrasound image obtained using a GE LOGIQ P7 system with a linear array transducer (4–12 MHz) demonstrates a well-circumscribed hypoechoic mass (black arrowheads) within the subcutaneous layer overlying the fourth metacarpal (4th MC). (**B**) On short-axis B-mode imaging, the mass (black arrowheads) lies superficial to the fourth and fifth metacarpals, displacing rather than infiltrating adjacent structures, with tissue planes preserved relative to the digital artery (DA). The underlying cortex is intact. The lesion is homogeneously hypoechoic without septations, calcifications, or cortical erosion, adopting a fusiform configuration consistent with a peripheral nerve sheath tumor [3,4,5,6]. This pattern of well-defined margins and preserved tissue planes preoperatively suggested the feasibility of complete resection. (**C**) Long-axis power Doppler image demonstrates mild internal vascularity (red arrowhead). Of greater diagnostic relevance, a hypoechoic linear structure with a fascicular appearance (white arrows) is identified overlying the fourth dorsal interosseous muscle (4th DI), raising the possibility of an adjacent nerve fascicle in continuity with the lesion. Its anatomical location strongly suggests involvement of the deep motor branch of the ulnar nerve, posing a potential risk to intrinsic hand function during resection. This appearance is consistent with the fascicular sign, a recognized sonographic feature of peripheral nerve sheath tumors [3,4]. Importantly, the absence of marked hypervascularity does not support malignant transformation [5,6]. (**D**) Short-axis power Doppler imaging also shows limited intralesional vascularity. The white boxs in the subgraphs indicate the transducer position.These findings, interpreted within the context of a confirmed NF1 diagnosis, supported a preoperative designation of localized neurofibroma. The fusiform morphology, fascicular sign, and limited vascularity observed here are precisely the sonographic features expected in NF1-associated localized neurofibromas, as opposed to the infiltrative plexiform subtype or malignant peripheral nerve sheath tumor [5]. Beyond diagnosis, this case illustrates how systematic sonographic assessment directly shapes operative strategy: the preserved tissue planes and limited vascularity informed preparation for complete resection, while the possibility of fascicular involvement prompted anticipatory readiness for nerve repair. When any preoperative concern remains, including infiltrative margins, marked hypervascularity, or deep extension, additional magnetic resonance imaging (MRI) is recommended prior to surgical intervention [3,7,8,9].

**Figure 3:**
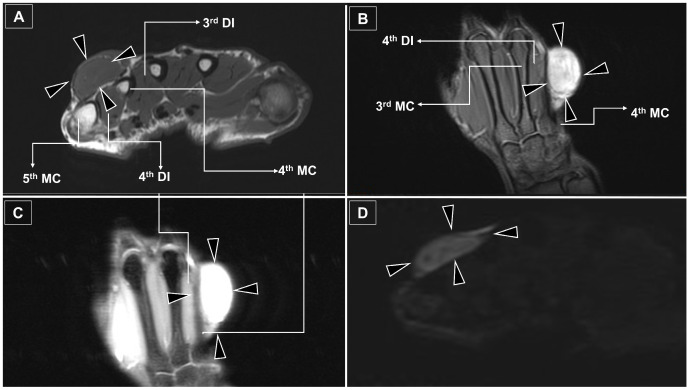
MRI of the Dorsal Hand Mass with Differential Diagnostic Considerations. (**A**) Axial T1-weighted image demonstrates a well-circumscribed subcutaneous mass (black arrowheads) isointense to the adjacent dorsal interosseous muscle. (**B**) Coronal fat-suppressed T1-weighted image shows persistent T1 hyperintensity relative to adjacent muscle (black arrowheads), suggesting proteinaceous or myxoid content. (**C**) Coronal T2-weighted image demonstrates marked T2 hyperintensity (black arrowheads), consistent with a peripheral nerve sheath tumor [9]. (**D**) Axial diffusion-weighted imaging shows focal diffusion restriction (black arrowheads), a variable finding that does not reliably distinguish benign from malignant peripheral nerve sheath tumors [8]. In the present case, the combination of well-defined margins, subcutaneous location, persistent T1 hyperintensity on fat-suppressed sequences, and marked T2 hyperintensity, in the absence of aggressive imaging features, favors a benign peripheral nerve sheath tumor, with neurofibroma included in the differential diagnosis (as NF1). Given that peripheral nerve sheath tumors are uncommon in the hand, accounting for approximately 1.8–4.9% of hand tumors when traumatic neuromas are excluded, careful differential diagnosis remains essential. However, an important limitation persists: MRI cannot reliably distinguish neurofibroma from schwannoma, nor can it definitively exclude malignant transformation [8]. To address this, we propose that a dual-modality approach (ultrasound and MRI) ensures the most comprehensive preoperative assessment for NF1-related hand tumors. While ultrasound offers superior real-time detail for tracing small nerve branches, MRI provides essential structural depth and soft-tissue characterization. Ultimately, despite these imaging advancements, histopathology remains the gold standard for definitive diagnosis. Clinicians must remain alert for malignant features, such as infiltrative margins, deep extension, osseous destruction, perilesional edema, and marked internal heterogeneity [5,10,11]. In this case, the surgical indication was based on a multifaceted assessment: progressive neurologic symptoms, a documented increase in tumor size over three years, and the necessity to rule out malignant transformation—a well-recognized risk in the NF1 population. Beyond these clinical factors, the psychosocial burden arising from the tumor’s appearance also factored into the decision for definitive excision.

**Figure 4:**
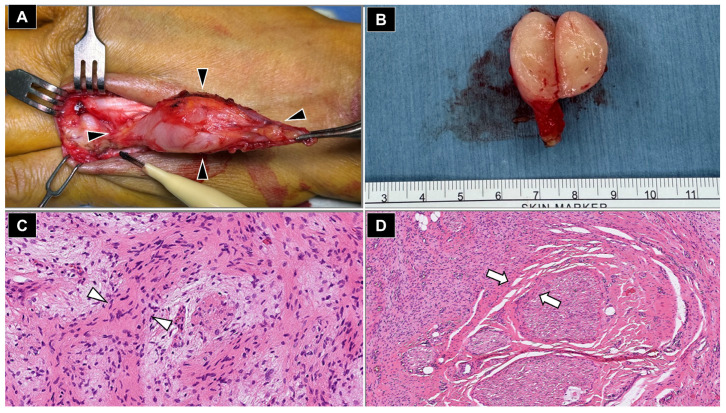
Intraoperative and Histopathological Findings of the Resected Dorsal Hand Tumor. (**A**) Intraoperative photograph showing a well-circumscribed, spindle-shaped mass (black arrowheads) intimately associated with the dorsal digital nerve, consistent with a nerve sheath tumor origin. (**B**) Gross specimen revealing an ovoid, encapsulated tumor with dimensions as illustrated, with a homogeneous, tan-white solid cut surface. (**C**) High-power hematoxylin and eosin section showing interlacing bundles of spindle-shaped cells with fibroblastic morphology (white arrowheads) within a myxoid stroma, characteristic of neurofibroma. (**D**) Low-power section illustrating infiltrative growth with entrapment of native nerve fascicles (white arrows), a pathognomonic feature distinguishing neurofibroma from schwannoma. Despite a gross appearance suggestive of schwannoma—i.e., a well-defined contour, ovoid shape, and homogeneous cut surface—the definitive distinction lies at the histologic level. Schwannomas are truly encapsulated tumors that arise eccentrically from a single nerve fascicle, whereas neurofibromas lack a true capsule and diffusely infiltrate the parent nerve, incorporating Schwann cells, perineural fibroblasts, and axons within an abundant myxoid matrix [2,9]. The entrapped nerve fascicles illustrated in (**D**) demonstrate an infiltrative growth pattern that significantly elevates the risk of iatrogenic nerve injury. Consequently, a straightforward tumor excision was deemed unfeasible, necessitating a more meticulous microsurgical dissection to preserve nerve integrity. Taken together, the combination of infiltrative architecture, mixed spindle cell proliferation, myxoid stromal change, and the established clinical diagnosis of NF1 confirms the final diagnosis of a solitary neurofibroma of the hand [2,10].

**Figure 5:**
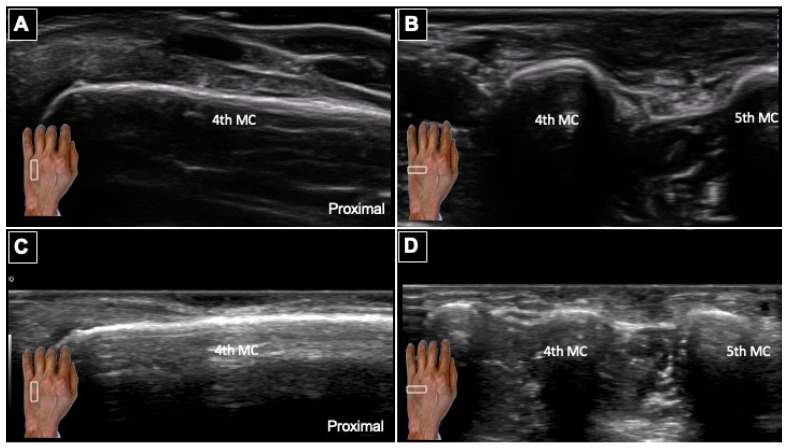
Postoperative Ultrasonographic Follow-up at 3 and 12 Months after Excision. (**A**) Long-axis and (**B**) short-axis high-resolution ultrasonographic views obtained at 3 months show no evidence of residual or recurrent hypoechoic mass within the operative field. (**C**) Long-axis and (**D**) short-axis views at 12-month follow-up similarly demonstrate no sonographic evidence of local tumor recurrence, nodular lesion, or abnormal enlargement of adjacent digital nerve structures. The white boxes in the subgraphs indicate the transducer position. All follow-up examinations were performed by the same experienced sonologist. Postoperative recovery was uneventful, with preserved hand motor function and fine motor performance. Postoperatively, the patient reported mild localized paresthesia adjacent to the incision, which was attributed to the sacrifice of a dorsal digital branch during tumor excision. This case illustrates how structured sonographic surveillance can be integrated into the long-term management of NF1-associated peripheral nerve tumors. Patients with NF1 carry a lifetime risk of malignant peripheral nerve sheath tumor of approximately 8–13%, frequently arising from pre-existing neurofibromas [2]. Accordingly, postoperative monitoring requires not merely confirmation of resection adequacy, but ongoing vigilance for interval morphological change. Although MRI remains the reference standard for deep-seated lesions or suspected malignant transformation [6,9,12], high-resolution ultrasonography enabled reliable longitudinal assessment in this case. Further, it provided prospective data supporting its utility for detecting peripheral nerve changes in NF1 patients over time [8]. Preoperatively, sonographic mapping assisted in incision planning and evaluation of adjacent extensor structures; postoperatively, serial imaging confirmed clearance throughout the first postoperative year. These findings support the emerging role for ultrasonography as a practical tool for both surgical planning and postoperative surveillance in superficial NF1-related nerve tumors [4,8,13].

## Data Availability

The original contributions presented in the study are included in the article; further inquiries can be directed to the corresponding author.

## Abbreviations

The following abbreviations are used in this manuscript:

NF1	Neurofibromatosis type 1
MRI	Magnetic resonance imaging
DWI	Diffusion-weighted imaging
MPNST	Malignant peripheral nerve sheath tumor
HRUS	High-resolution ultrasonography
DA	Digital artery
DI	Dorsal interosseous muscle
MC	Metacarpal
H&E	Hematoxylin and eosin
